# Visceral Leishmaniasis in Traveler to Guyana Caused by *Leishmania siamensis*, London, UK

**DOI:** 10.3201/eid2401.161428

**Published:** 2018-01

**Authors:** Spencer D. Polley, Julie Watson, Peter L. Chiodini, Diana N.J. Lockwood

**Affiliations:** Health Services Laboratories LLP Analytics, London, UK (S.D. Polley, J. Watson):; Hospital for Tropical Diseases, University College London Hospitals NHS Foundation Trust, London (P.L. Chiodini, D.N.J. Lockwood);; London School of Hygiene and Tropical Medicine, London (P.L. Chiodini, D.N.J. Lockwood);; National Institute of Health Research, University College London Hospitals Biomedical Research Centre, London (P.L. Chiodini)

**Keywords:** Leishmania siamensis, parasites, visceral leishmaniasis, zoonosis, phlebotomine sand fly, Sergentomyia (Neophlebotomus) gemmea, anthropophilic, zoophilic, authochthonous, hepatosplenomegaly, cervical lymphadenopathy, sequence typing, sandflies, zoonoses, Guyana, South America, Thailand

## Abstract

The parasite *Leishmania siamensis* is a zoonotic agent of leishmaniasis; infection in animals has been documented in Europe and the United States. Reported authochthonous human infections have been limited to Thailand. We report a case of human visceral *Leishmania siamensis* infection acquired in Guyana, suggesting colonization in South America.

A 65-year-old woman was admitted to a hospital in London, UK during March 2014 after collapsing in the street. She was anemic and mildly thrombocytopenic (hemoglobin level 8.1 g/dL, leukocyte count 4.62 × 10^9^/L, platelet count 143 × 10^9^/L). She had been unwell for 14 months, experiencing night sweats and a steady loss of energy but was otherwise asymptomatic. She reported no fever. On examination, she had hepatic enlargement and lymphadenopathy, was afebrile, and had normal liver function test results. She also had negative serologic test results for HIV, hepatitis B, hepatitis C, and the parasitic nematode spp. *Strongyloides*. Her CD4 count was 790 and rheumatoid factor was weakly positive; her immunoglobulin levels were within reference ranges. 

The patient was from Guyana and migrated to the United Kingdom in 1967. Her relevant travel history comprised 2 recent visits to Guyana (Georgetown in 2012 and Freetown in 2013), Caribbean Grenada in 2012, Ghana in 2005, and France (Paris and Marseille) in 2003. We investigated for hematologic malignancy by bone marrow aspiration; *Leishmania* amastigotes were visible.

Results of serologic testing for *Leishmania* antibodies were negative by using a Rapydtest (rK39 RDT; Apacor Ltd., Berkshire, UK) and weakly positive by using the direct agglutination test (1:3,200; cutoff 1:1,600). *Leishmania* DNA was extracted from the sample. *Leishmania*–specific PCR amplification produced positive amplicons from kinetoplast minicircle, genomic heat shock protein 70, and internal transcribed spacer 1 (ITS1) targets. The kinetoplast–specific amplicon size appeared nearest to that of *Leishmania major*, which is not considered an agent of visceral leishmaniasis among humans.

The size and restriction fragment length polymorphism banding pattern of the ITS1 amplicon was distinct from all previously sampled human *Leishmania* species. Sequencing of the ITS1 amplicon revealed either 99% or 100% identity to ITS1 sequences from *Leishmania siamensis*. Lower levels of identity were seen in homologs from other human *Leishmania* species. Phylogenetic analysis of these sequences against reference sequences from other *Leishmania* species confirmed they clustered with *L. siamensis* sequences as a monophyletic group, supported by bootstrap values of 100% (Figure). We saw a major divergence from other *Leishmania* sequences.

**Figure:**
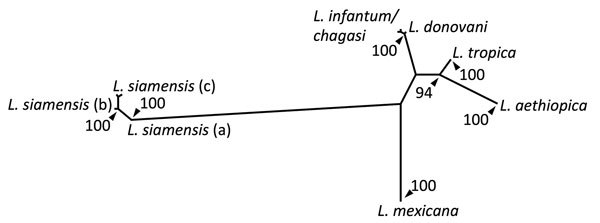
Consensus distance–based tree generated from the infecting amastigote’s internal transcribed spacer 1 sequence and homologous sequences from other related human *Leishmania*–infected samples. Posterior bootstrap values are presented as the percentage of trees from 100 pseudorandomly sampled datasets which supported a given node with a value >90%. The sequences for the various terminal nodes, chosen for nearest identity to the derived sequence (EMBL-LT577674) by a BLASTn search are as follows: *L. aethiopica*, GQ920674, GQ920676, GQ920673; *L. tropica*, FJ948454, FJ948450, FJ948456; *L. mexicana*, AJ00313, AF466381; *L. donovani*, FJ753386, AM901452, AM901453, *L. infantum/chagasi* GU045592, FN398343, GU045591; *L. siamensis* (a), EF200012, (b), JX195637, GQ28127, JQ617283, JQ001751, GQ293226, (c), JQ866907, GQ226034. Branch lengths are proportional to the intersequence divergence, calculated by using the Fitch-Margoliash method of measuring pairwise distances derived from the F84 model.

We treated the patient with liposomal amphotericin B (AmBisome [Gilead Sciences Ltd., London, UK) at a dose of 3 mg/kg given on days 1–5, and 7-day courses beginning on days 10 and 20. She responded well and her blood test results returned to reference values. She had some mild reversible renal impairment during treatment; she recovered and did not relapse during a 10-month follow-up period.

Autochthonous human visceral leishmaniasis caused by *L. siamensis* was thought to be geographically confined to Thailand (*1*–*5*). Many of those case-patients also showed evidence of immune deficiency, such as HIV infection (*1*,*3*). This patient had no evidence of immune deficiency, nor had one manifest during the follow-up period after her illness, yet she had a negative rK39 test result (which detects *Leishmania* antibodies) despite a visceral infection. Another patient with *L. siamensis* visceral leishmaniasis also had a negative rK39 test result (*2*); therefore, *L. siamensis* infections may not be detectable by rK39 testing.

The phlebotomine sand fly *Sergentomyia* (*Neophlebotomus*) *gemmea* is a possible vector for *L. siamensis* in Thailand (*6*–*8*). Sand flies from the *Sergentomyia* genus are generally zoophilic and therefore discounted as vectors of medically consequential *Leishmania* spp. Several *Sergentomyia* species are present in Europe and in South and North America, which may explain the presence of autochthonous zoonotic *L. siamensis* in these locations. Human infection with *L. siamensis* outside Thailand raises questions concerning transmission of this species to humans by anthropophilic phlebotomine sand flies or other species generally categorized as zoophilic.
